# Methods for calculating Protection Equality for conservation planning

**DOI:** 10.1371/journal.pone.0171591

**Published:** 2017-02-15

**Authors:** Alienor L. M. Chauvenet, Caitlin D. Kuempel, Jennifer McGowan, Maria Beger, Hugh P. Possingham

**Affiliations:** 1 Centre for Biodiversity and Conservation Science, School of Biological Sciences, University of Queensland, St Lucia, Queensland, Australia; 2 ARC Centre of Excellence for Environmental Decisions, University of Queensland, St Lucia, Queensland, Australia; 3 School of Biology, University of Leeds, Leeds, West Yorkshire, United Kingdom; 4 The Nature Conservancy, South Brisbane, Queensland, Australia; University of Waikato, NEW ZEALAND

## Abstract

Protected Areas (PAs) are a central part of biodiversity conservation strategies around the world. Today, PAs cover *c*15% of the Earth’s land mass and *c*3% of the global oceans. These numbers are expected to grow rapidly to meet the Convention on Biological Diversity’s Aichi Biodiversity target 11, which aims to see 17% and 10% of terrestrial and marine biomes protected, respectively, by 2020. This target also requires countries to ensure that PAs protect an “ecologically representative” sample of their biodiversity. At present, there is no clear definition of what desirable ecological representation looks like, or guidelines of how to standardize its assessment as the PA estate grows. We propose a systematic approach to measure ecological representation in PA networks using the Protection Equality (PE) metric, which measures how equally ecological features, such as habitats, within a country’s borders are protected. We present an R package and two Protection Equality (PE) measures; proportional to area PE, and fixed area PE, which measure the representativeness of a country’s PA network. We illustrate the PE metrics with two case studies: coral reef protection across countries and ecoregions in the Coral Triangle, and representation of ecoregions of six of the largest countries in the world. Our results provide repeatable transparency to the issue of representation in PA networks and provide a starting point for further discussion, evaluation and testing of representation metrics. They also highlight clear shortcomings in current PA networks, particularly where they are biased towards certain assemblage types or habitats. Our proposed metrics should be used to report on measuring progress towards the representation component of Aichi Target 11. The PE metrics can be used to measure the representation of any kind of ecological feature including: species, ecoregions, processes or habitats.

## Introduction

Protected Areas (PAs) are a central part of biodiversity conservation strategies around the world. There are currently more than 200,000 PAs under International Union for Conservation of Nature (IUCN) designation that cover *c*.15% of the Earth’s land mass [1–3], and *c*.3% of the global oceans [4]. A sharp increase in those numbers, especially in the sea, is expected in the coming years as countries that signed the Convention on Biological Diversity (CBD) aim to protect 17% and 10% of terrestrial and marine jurisdictions respectively, by 2020 (Aichi Target 11, [5]). Moreover, the CBD states these targets should be achieved through “effectively and equitably managed, *ecologically representative* and well-connected systems of protected areas” [5]. At present, there are no globally accepted metrics that evaluate how well systems of PAs meet all these objectives.

Ecological representation is a key principle of systematic conservation planning that broadly aims to ensure a PA system protects a sample of all biodiversity present [6]. Usually, this is accomplished through setting quantitative targets for individual conservation features (e.g. species or habitats) to be protected. Despite some criticisms (e.g. the arbitrariness in target amounts [7, 8]), target-based conservation planning is now commonplace and driving biodiversity commitments at both global and national levels. However, questions remain regarding the adequacy of target amounts and comparability between countries. For example, what biodiversity outcomes do we achieve with 17% protection of terrestrial habitats? Does it make sense to protect an equal percentage of ecologically or geo-politically defined units, such as ecoregions? What is good ecological representation, and how should we measure it?

Barr *et al*. [9] identified the need for a comparable measure of ecological representation between countries as part of a systematic conservation planning approach. The authors adapted the Protection Equality (PE) metric from the Gini coefficient [10] and introduced it as a way to determine the level of representation of a PA network. They discovered that it was more independent of the total area protected than other common measures of representation. The Gini coefficient is an index used in economics to measure the difference between how a perfectly equitable distribution of individual income accumulates and the actual distribution of income (called the Lorenz curve, [11]). It has been adapted to measure inequality in various others fields, such as: education [12], size hierarchy in plant populations [13], the use of carbon sources in bacterial soil communities [14], and access of urban residents to green space [15]. However, until the recent work by Barr and colleagues [9] it had not been applied to issues of conservation concern, besides investigating changes to income following a new conservation policy (e.g. [16–18]). As part of the PA evaluation toolkit under Aichi Target 11, the PE metric could allow for comparisons of representation across different countries’ PA systems, especially as total area increases rapidly under international agreements for biodiversity protection.

Barr and colleagues [9] proposed a PE metric that measures ecological representation of conservation features as the “proportional amount” of each feature under protection. For example, if 10% of each ecoregion is conserved, we achieve perfectly equitable representation. An alternative approach would be to protect a “fixed amount” of each feature (e.g. 1000 km^2^ of each ecoregion). These two approaches embody distinct policies under the representation component of Aichi Target 11 with very different outcomes for overall biodiversity. Here, we extend the work of Barr et al. [9] and compare the outcome of measuring protection using both ecological representation approaches using two case studies: (1) coral reef habitat protection in the Coral Triangle Marine Protected Area System (CTMPAS) and (2) large scale patterns of terrestrial protection in six of the world’s largest countries.

We present a refined measure of the PE metric proposed by Barr et al. (proportional PE) and propose an additional way to calculate PE (fixed area PE). Moreover, the Gini coefficient, on which the PE metric is based, was built using large numbers of habitats or ecosystem types. This leads to an over-inflation of the PE metric for smaller numbers of conservation features. Hence, we also introduce a correction factor for PE. The two PE versions with the correcting factor are available as part of a new R package called *ProtectEqual* [19] (see S1 Appendix).

## Materials and methods

### Mathematical formulation

#### Protection Equality metric (PE)

Consider a region of interest (e.g. a country, a state or a continent) of total area *a*_*tot*_, which contains *N* conservation features, indexed by the subscript *i*, with *i* ∈ Z^+^. Each conservation feature *i* has an area *a*_*i*_ inside the region of interest such that:

$$a_{tot}=\overset{N}{\underset{i=1}{\sum }}a_{i}\;.$$

Let the amount of conservation feature *i*, which is designated as a Protected Area (PA) be denoted *p*_*i*,_ with 0 < *p*_*i*_ < *a*_*i*_.

We rank the level of protection of conservation features in ascending order (either fixed area protection *p*_*i*_ (in ha for example) or proportion of the ecoregion protected *p*_*i*_/*a*_*i*_) and calculate the cumulative level of protection of conservation features *y*_*i*_ for *i* = 1,2,…*N*, using

$${\text{y}}_{i}=\overset{i}{\underset{j=1}{\sum }}p_{j}\;\;or\;\;{\text{y}}_{i}=\overset{i}{\underset{j=1}{\sum }}\frac{p_{j}}{a_{j}}\;.$$

**Fig 1:**
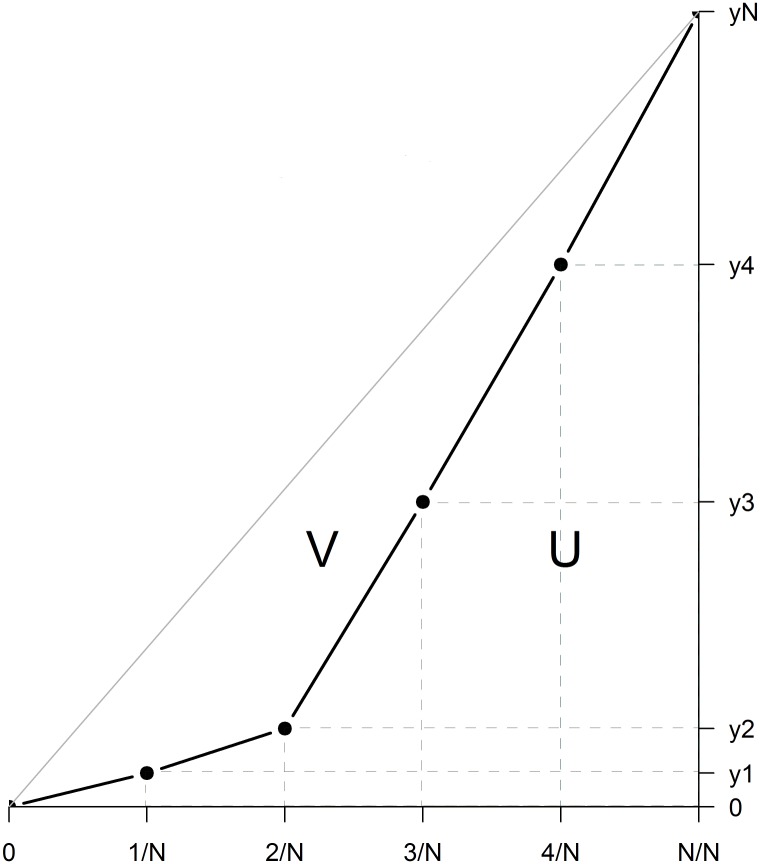
Illustration of how Protection Equality is calculated (here *N* = 5). The line of perfect equality is shown in grey. The black curve is equivalent to the Lorenz curve and formed by the cumulative level of protection of each ecoregion *i* against *i*/*N*, with 1 ≤ *i* ≤ *N*. On the y-axis, y1 to y*N* are either cumulative fixed area protection (p_*i*_) or cumulative proportional protection (p_*i*_ /a_*i*_). The dashed lines highlight the triangles and rectangles used to approximate area U, with PE calculated as the ratio of U over U+V.

To estimate PE, we plot *y*_*i*_ against *i*/*N* for i = 1,2,…*N* (illustrated in Fig 1). PE is then calculated by estimating the area under the curve formed by the protection of the conservation features (U; Fig 1; equivalent to the Lorenz curve) divided by the total area under the line of perfect equality (U+V; Fig 1).

$$PE=\frac{U}{U+V}\;.$$

The steps to derive formulas for fixed area (PE_*f*_) and proportional (PE_*p*_) are detailed in S2 Appendix. Each PE metric can be calculated as follows:

$${\text{PE}}_{f}=\frac{\frac{1}{N}\times \left(\frac{1}{2}\sum_{i=1}^{N}p_{i}+\;\sum_{i=1}^{N-1}p_{i}\times \left(N-i\right)\right)}{\frac{1}{2}\times \sum_{i=1}^{N}p_{i}}\;\;;$$

$${\text{PE}}_{p}=\frac{\frac{1}{N}\times \left(\frac{1}{2}\sum_{i=1}^{N}\frac{p_{i}}{a_{i}}+\;\sum_{i=1}^{N-1}\frac{p_{i}}{a_{i}}\times \;\left(N-i\right)\right)}{\;\frac{1}{2}\times \sum_{i=1}^{N}\frac{p_{i}}{a_{i}}}\;\;.$$

#### Corrected Protection Equality metric

When the number of conservation features *N* is small, the worst value that can be obtained for PE, even with maximum inequality, is substantially greater than 0. For example, imagine a region of interest with *N* conservation features with only one of them with some protection but none for the others, which would represent the greatest possible inequality in protection. When there are two conservation features, the minimum PE is 1/2 and when there are three then PE is 1/3 even though you would expect a country or region with complete inequality of conservation features to have a PE of 0 (S1 Fig). As *N* increases, however, (e.g. *N* = 100) PE tends to 0 (S1 Fig and S1 Table). To solve this problem, we created a correction factor, which rescales PE between 0 and 1 when *N* is small.

$${\text{PE}}_{c}=\left(\text{PE}-\frac{1}{N}\right)*\left(\frac{N}{N-1}\right)\;.$$

PE_*c*_ is lower than PE for small *N* values but tends to PE as *N* increases (both fixed and proportional PE). Under perfect inequality corrected PE_*f*_ and PE_*p*_ now always equal zero. We used the corrected PE formulation in the following analysis.

#### Simulations

With two possible ways of measuring PE, the first question is, how and why do they differ? We compared the performance of PE_*f*_ and PE_*p*_ under two different scenarios. Scenario 1: when 10% of all ecoregions in a country/region are protected (hence PE_*p*_ is equal to 1), how does PE_*f*_ perform as the variability in ecoregion size increases? Scenario 2: when 100 ha of all ecoregions in a country/region are protected (hence PE_*f*_ is equal to 1), how does PE_*p*_ perform as the variability in ecoregion size increases? We simulated this for 5, 10, 100 and 1000 ecoregions, and tested the two different PE metrics when areas of all ecoregions in a country/region were identical and when they differed in size.

### Case studies

#### Case study 1: Representation of coral reef habitat protection in the CTMPAS

The Coral Triangle encompasses six nations: Indonesia, Malaysia, Philippines, Papua New Guinea, Solomon Islands, and Timor Leste and is considered the epicentre of the world’s marine tropical biodiversity [20, 21]. These countries are signatories to the Coral Triangle Initiative on Coral Reefs, Fisheries and Food Security (CTI-CFF, [22]), a multilateral partnership focused on improving coral reef sustainability and biodiversity in the region, with a focus on livelihoods and food security for the 120 million people dependent on coral reef ecosystems for sustenance, income and cultural identity [22]. Seascape management, which includes marine protected areas (MPAs), is an important component of the CTI-CFF. The Coral Triangle encompasses 21 distinct biogeographic ecoregions [23]. These ecoregions are important as they act as ecological jurisdictions that represent species assemblage turnover, providing a non-political stratification system to evaluate levels of PE across the region, beyond country borders.

In line with the CTI-CFF’s aim to develop a representative network of MPAs, Beger and colleagues [24] conducted a gap analysis for both ecoregion and country level coral reef protection. Using the most up to date boundaries of MPAs available from the Coral Triangle Atlas (http://ctatlas.reefbase.org/) and partners across the region (2013), reef habitats were intersected with MPA boundaries in ArcGIS to determine the amount of coral habitat offered any kind of protection, and the amount protected within estimated no-take areas. Given that the level of enforcement across MPAs is difficult to verify for this region all MPAs were treated equally with respect to their level of protection when calculating the area of an ecoregion protected, *p*_*i*_. Beger and colleagues [24] acknowledge this approach is subject to estimation errors for the true amount of protected habitats [21, 25]. Additional data processing methods, assessment, and geoprocessing rules are described in [24] for the coral habitat and MPA dataset. Given that this work formally precedes expanding conservation efforts in the region, it is an ideal case study to evaluate our PE metrics.

We calculated the fixed area and proportional PE of coral habitat within the Coral Triangle region. We investigated two levels of grouping: first PE_*p*_ and PE_*f*_ were calculated across countries (i.e. using amount of coral habitat protected in each country; *N* = 6); second PE_*p*_ and PE_*f*_ were calculated across ecoregions (i.e. using amount of coral habitat protected in each ecoregion regardless of the country; *N* = 21). For each grouping we also recorded the total *p*_*i*_ and *a*_*i*_, the average proportion of coral within the areas (*p*_*i*_/*a*_*i*_), and the number of countries or ecoregions which offered some level of coral protection (*p*_*i*_ >0)

#### Case study 2: Large scale patterns of representation

We investigated PE for the six largest countries in the world for which >90% of their PAs had well-defined boundaries in the World Database on Protected Areas (WDPA; accessed December 2016, [26]) in order to illustrate how our two measures of PE compare at a large scale. Only terrestrial PAs, with a designated IUCN category I-IV (i.e. those managed primarily for biodiversity), were considered in our analysis. Based on the above criteria, the six countries were Argentina, Australia, Brazil, Canada, Democratic Republic of (DR) Congo, and the United States. It is worth noting that although China and Russia are bigger than Argentina and DR Congo, only 10.5% and 16.4% of their respective PAs had clearly delineated boundaries in the WDPA database; the rest were only recorded as a point location. As a result, we discarded both countries from the analysis. All selected countries are larger than two million square kilometres.

We used the 825 ecoregions developed by the World Wildlife Fund (WWF) [27] to divide each country into ecoregions; the ecoregions “Lakes”, “Rock and Ice” and “Mangroves” were excluded to account for spatial mismatches between countries [9]. PA coverage was estimated using the 2015 version of the WDPA database and spatial processing suggestions from UNEP-WCMC [28]. Total area (*a*_*i*_) and total protected area of each ecoregion within each country (*p*_*i*_) were extracted using ArcGIS (version 10.2) by intersecting three layers: an equal-area projection of countries, the WWF ecoregions, and the WDPA terrestrial PAs. All countries had 17 or more ecoregions.

We calculated the proportional and fixed area PE of ecoregions within all six countries. For each country, we recorded both PE_*p*_ and PE_*f*_, total protected area *p*_*i*_, mean *p*_*i*_ and *a*_*i*_ across all ecoregions, the mean and median *p*_*i*_ / *a*_*i*_ across all ecoregions, the percent of the country under protection as well the number of ecoregions with more than zero area protected.

## Results

### Simulation

**Fig 2:**
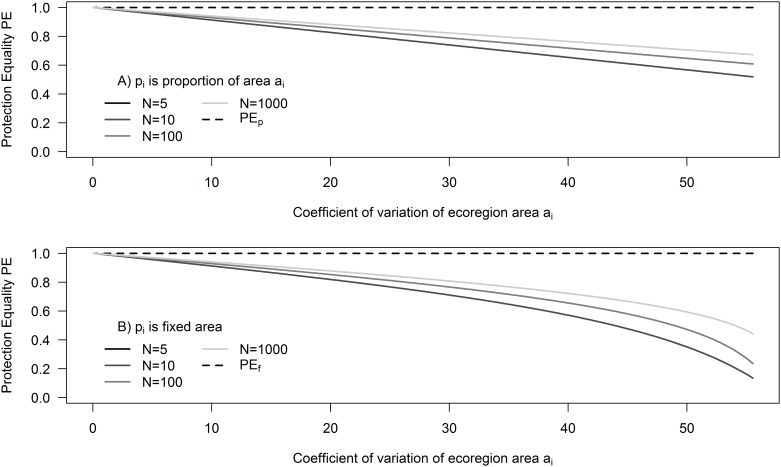
Results of simulation of Protection Equality (PE) under two scenarios as a function of the coefficient of variation of ecoregion area *a*_*i*._ (A) When ecoregions are protected at fixed proportion of *a*_*i*_, e.g. 5%, and (B) When ecoregions are protected at a fixed area, e.g. 100 ha. We simulated 4 different numbers of ecoregions: 5, 10, 100, 1000.

If all conservation features have the same size, *a*_*i*_ = *C*_*a*_ ∀ *i*, and each has the same area protected *p*_*i*_ = *C*_*p*_ ∀ *i*, where *C*_*a*_ and *C*_*p*_ are constant, then we know PE_*f*_ = PE_*p*_ = 1. We thus might expect PE_*f*_ and PE_*p*_ to change as the variability in the size of conservation features increases. We assessed the values of PE_*f*_ and PE_*p*_ when conservation features are protected in the same proportion *p*_*i*_ = *Ca*_*i*_ where *C* is a constant (scenario 1 where PE_*p*_ is always 1) and when conservation features are protected by a fixed amount *p*_*i*_ = *C* (scenario 2 where PE_*f*_ is always 1) respectively, as a function of the coefficient of variation of the area, *a*_*i*_, of all conservation features in a region (Fig 2). As the coefficient of variation of *a*_*i*_ increases (i.e. there is a larger disparity between the size of all the conservation features), both of the PE metrics decrease (measured as PE_*f*_ for scenario 1, and PE_*p*_ for scenario 2). PE_*p*_ decreases much faster than PE_*f*_ as a function of variation in *a*_*i*_ for their respective scenarios, although the difference between the two measures is small up to a coefficient of variation of *a*_*i*_ of *c*. 20% (S2 Fig). This implies that countries aiming for protection proportional to *a*_*i*_, can achieve a higher representation score than countries aiming for a fixed level of protection, regardless of the metric used to measure representation. However, this difference only matters if the country’s ecoregions have large differences in size; it is also more pronounced when *N* is small.

Thus, countries with only a few ecoregions can achieve better proportional and fixed area PE by aiming to protect an equal percentage of each ecoregion, unless ecoregions are similar in size. If countries have many ecoregions, they are less affected by this pattern and can reach high PE by either protecting a set percentage or a set amount of each ecoregion. As a result, PE_*p*_ and PE_*f*_ values should not be contrasted with each other and countries should be compared using the same metric.

### Case studies

#### Case study 1: Representation of the coral reef habitat protection in the CTMPAS

**Fig 3:**
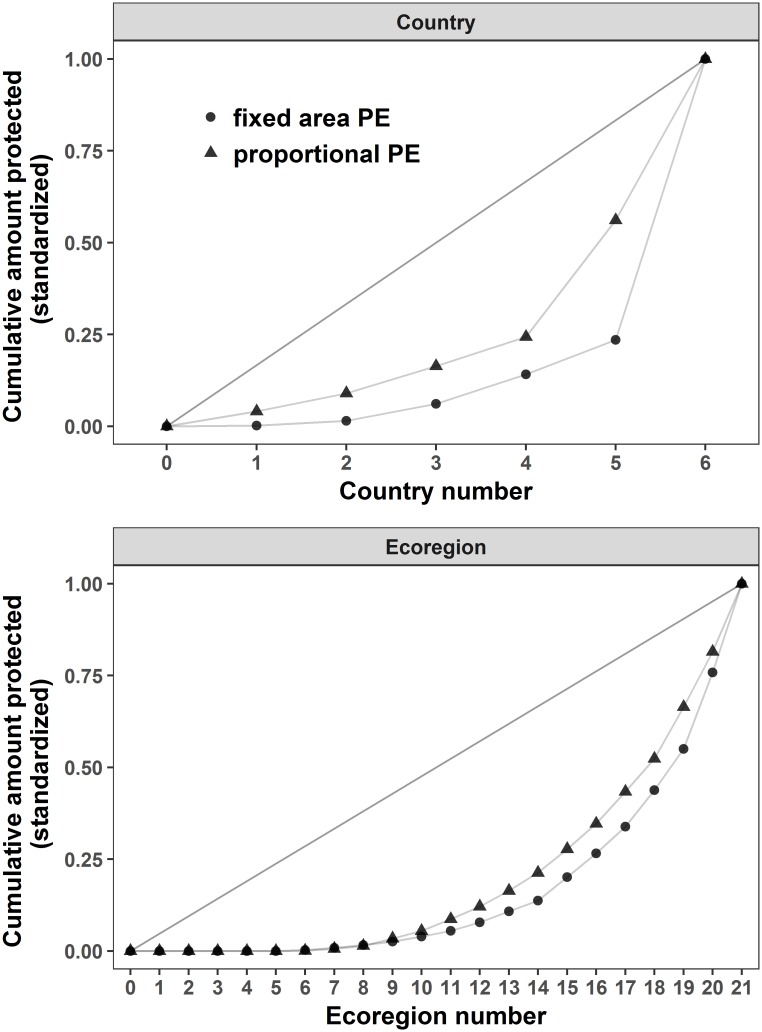
Coral reef habitat Protection Equality (PE) within countries and ecoregions in the Coral Triangle region. The graphs are displayed on the standardized scale. Corresponding PE values are given in Table 1 and were calculated using the PE_*p*_ (triangles) and PE_*f*_ (dots) metrics.

**Fig 4:**
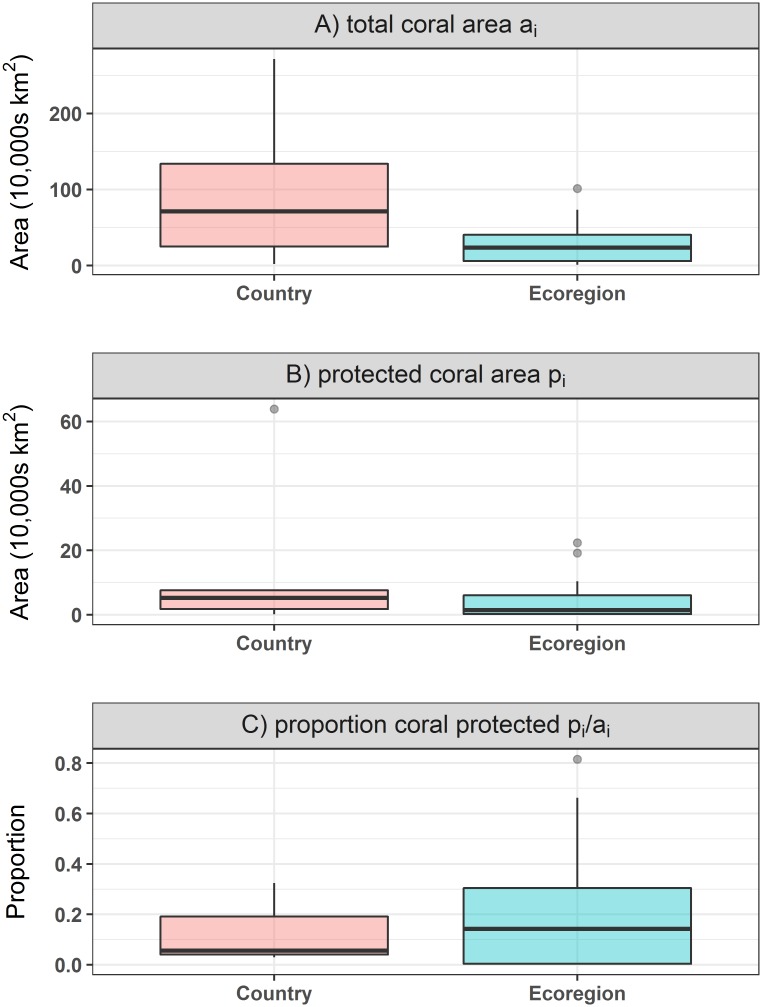
Boxplot of the (A) total coral area (a_*i*_), (B) protected coral area (p_*i*_) and (C) proportion of coral protected (p_*i*_/ a_*i*_) in the coral triangle within countries and within ecoregions. Shown is data for six countries and 21 ecoregions.

At both the country and ecoregion level, the PE values for the Coral Triangle were relatively low (all <0.44; Table 1; Figs 3 and 4), indicating unequal representation of coral reef habitat protection among countries and among ecoregions.

**Table 1:** Proportional (PE_*p*_) and fixed area (PE_*f*_) Protection Equality of coral reef habitat in the Coral Triangle region, for its six countries, and its 21 ecoregions.

Unit	PE_*p*_	PE_*f*_	Total *p*_*i*_ (km^2^)	Total *a*_*i*_ (km^2^)	Mean (*p*_*i*_ / *a*_*i*_)	Median (*p*_*i*_ / *a*_*i*_)	Min (*p*_*i*_ /*a*_*i*_)	Max (*p*_*i*_ / *a*_*i*_)	*N*	Number of Units with > 0% coral protection (% of *N*)
**Country**	0.44	0.18	8356.52	58080.73	0.123	0.057	0.03	0.325	6	6 (100)
**Ecoregion**	0.38	0.30	9240.80	60769.04	0.210	0.142	0.00	0.816	21	17 (80.95)

At the country level, a much higher coral reef protection PE was achieved using the proportional PE than the fixed area PE, with PE_*f*_ being half the size of PE_*p*_ (Table 1; Figs 3 and 4). The very small PE_*f*_ value is explained by the fact that the amount of coral each country protected, *p*_*i*_, ranged from 12 to 6400 km^2^ across the six countries (i.e. two orders of magnitude) while the proportion of each country’s protected coral, *p*_*i*_/*a*_*i*_, ranged between 3 and 33%. This is indicative of the uneven distribution of coral reef habitat across countries. The six countries were therefore more equal in terms of proportional protection than fixed area protection of their coral reef habitats.

At the ecoregional level, the difference between PE_*f*_ and PE_*p*_ was much smaller (Table 1; Figs 3 and 4) with both values being less than 0.4. This implies that within ecoregions, the fixed and proportional level of protection of coral reef habitat is similar, although still quite small. The *p*_*i*_ values of all 21 ecoregions ranged from 0 to 2235.4 km^2^, which is a much smaller disparity than within countries. The proportional protection (*p*_*i*_/*a*_*i*_), however, spanned between 0 and 82%, which is a large range of values across 21 ecoregions. This difference explains why PE_*f*_ within ecoregions was higher than within countries, while PE_*p*_ within countries was higher than within ecoregions.

#### Case study 2: Large scale patterns of representation

**Fig 5:**
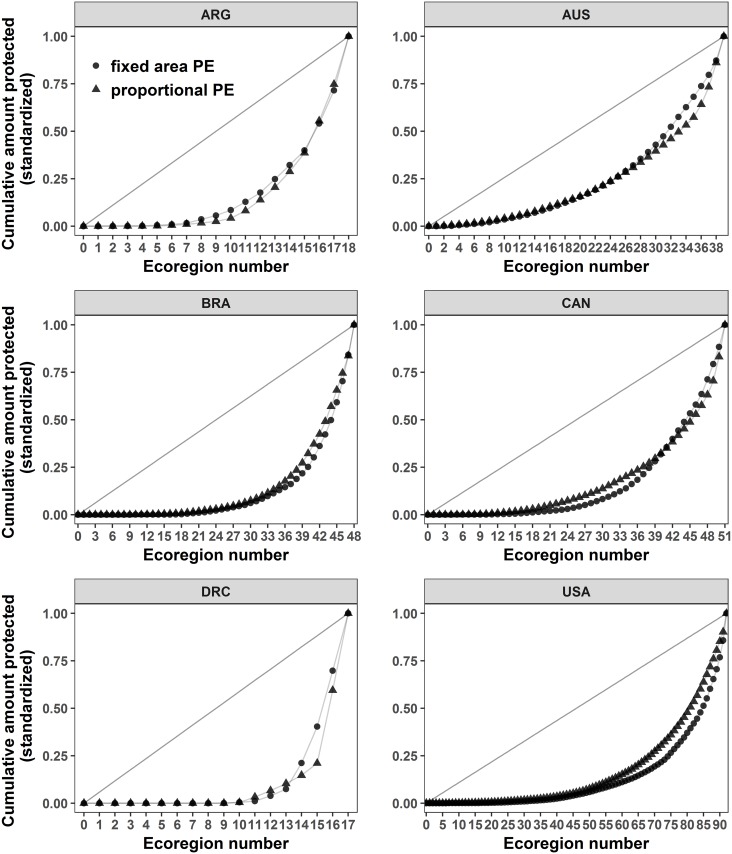
Protection Equality graph for the ecoregions of the six biggest countries in the world with > 90% of their PA clearly delimited in the WDPA database [26]. The graphs are displayed on the standardized scale. Corresponding PE values are given in Table 2 and were calculated using the PE_*p*_ (triangles) and PE_*f*_ (dots) metrics.

**Fig 6:**
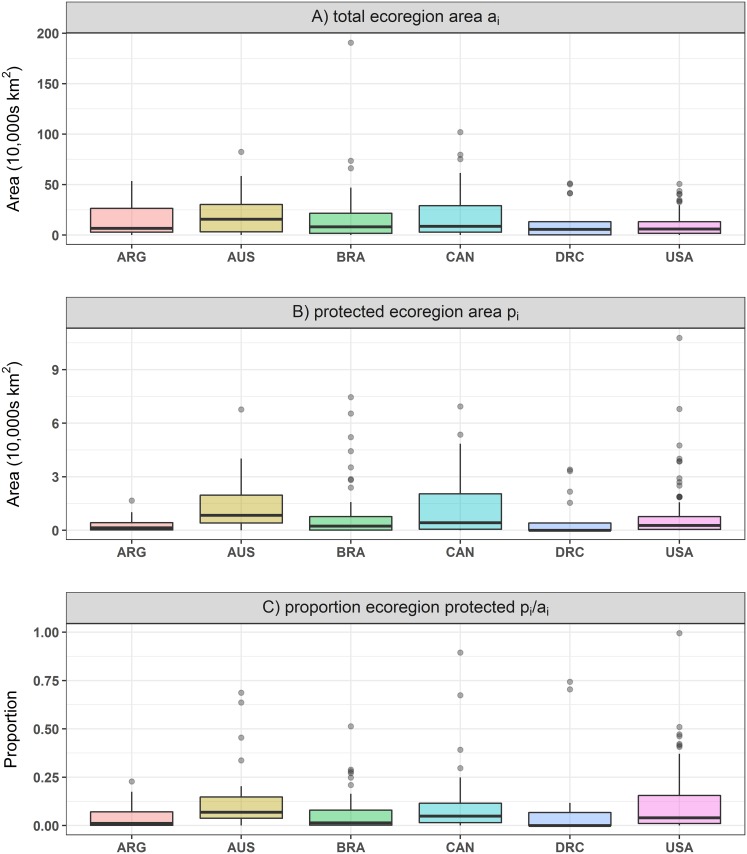
Boxplot of the (A) total ecoregion area, (B) protected ecoregion area and (C) proportion of ecoregion protected in the six largest countries: Australia (AUS), Argentina (ARG), Brazil (BRA), Canada (CAN), Democratic Republic of Congo (DRC) and United States of America (USA).

Similarly to our case study of the Coral Triangle, none of the six largest countries had a PE over 0.50, indicating a below average PE at the global scale (Table 2; Figs 5 and 6). Australia had the highest proportional and fixed area PE, while DR Congo had the lowest in both. Argentina and Brazil, and Canada and the USA, had similar fixed area PE values (PE_*f*_), respectively, despite all four countries having large differences in number of ecoregions. This shows that countries with more ecoregions can score similar PE to those with fewer ecoregions. Canada and the USA have near identical PE_*p*_, and similar mean percentages of protected area across ecoregions, yet the US has a much higher % of the country protected; this implies that either Canada is doing better than expected for its size or that the US is doing worse in terms of proportional protection (Table 2). Similarly for Argentina and Brazil, which have almost identical PE_*p*_ values, the latter shows a much higher mean *p*_*i*_ value than the former but they have similar mean *a*_*i*_ values. This indicates that Brazil, despite having more than twice as many ecoregions, protects a larger proportion of each on average than Argentina (Table 2). Surprisingly, DR Congo had a similar average proportion of ecoregion protected to Australia, Canada, and the US, but scored much lower for PE_*p*_. This could be explained by a large variance in *p*_*i*_ /*a*_*i*_ for DR Congo with several ecoregions with little or no protection, and a few with a very high protection percentage (Fig 6). Indeed, the median *p*_*i*_ /*a*_*i*_ for DR Congo is <1% (and only 53% of ecoregions have protection; Table 2), while for Australia, Canada and the USA, it is >4% (and > 94% of ecoregions have protection; Table 2).

**Table 2:** Proportional (PE_*p*_) and fixed (PE_*f*_) Protection Equality of the six largest countries in the world with > 90% of their PA delimited in the WDPA database [26].

Unit	PE_*p*_	PE_*f*_	Total *p*_*i*_ (km^2^)	Total *a*_*i*_ (km^2^)	Mean *p*_*i*_ (km^2^)	Mean *a*_*i*_ (km^2^)	Mean *p*_*i*_ / Mean *a*_*i*_	Mean (*p*_*i*_/*a*_*i*_)	Median (*p*_*i*_/*a*_*i*_)	Proportion Country protected	*N*	Number of Ecoregions with > 0% protection (%)
**Argentina**	0.29	0.32	58240	2782494	3236	154583	0.021	0.050	0.0113	0.021	18	18 (100)
**Australia**	0.46	0.48	531576	7710133	13630	197696	0.069	0.127	0.0692	0.069	39	38 (97.44)
**Brazil**	0.27	0.23	470958	8494125	9812	176961	0.055	0.066	0.0140	0.055	48	40 (83.34)
**Canada**	0.35	0.33	596057	9566050	11687	187570	0.062	0.104	0.0490	0.062	51	48 (94.12)
**DR Congo**	0.14	0.18	112662	2326621	6627	136860	0.048	0.108	0.0001	0.048	17	9 (52.94)
**United States**	0.34	0.27	759451	9254610	8255	100594	0.082	0.111	0.0404	0.082	92	88 (95.65)

## Discussion

In this paper, we build on the work of Barr *et al*. [9] to present two metrics to measure ecological representation of PA networks. The proportional (PE_*p*_) and fixed-area (PE_*f*_) PE metrics measure how equitably habitats are being represented within PA networks; the former looks at proportional protection—what fraction of each habitat is conserved, while the latter looks at absolute protection independent of how much of each habitat is available. We thus provide tools to compare countries in a systematic manner that is more informative than simply referring to the amount or percentage of area protected. Moreover, the open-access ProtectEqual R package [19] allows easy calculation of the two metrics. Thus, they can be incorporated in cost-benefit analyses as part of decision-making as managers can calculate the representation benefit of different actions, and knowing their cost, choose the most cost-efficient solution. We therefore believe that the PE metrics should be included in PA reporting under Aichi Target 11.

Our case studies demonstrate how PE_*f*_ and PE_*p*_ behave in real life situations, but also highlight their shortcomings. Neither metric is informative without contextual information such as, the available area for protection, or mean and median protection rates. PE should be used as part of a wider range of metrics to measure PA effectiveness. It could also be extended in several ways. First, ecoregions are a coarse classification of habitat types and within them we expect some habitats to be more degraded than others. Land cover classification could be used to refine the measure of habitat available (*a*_*i*_) as this value may be much smaller than the total ecoregion area; it can help determine whether there is any habitat left within the ecoregion that is suitable to put under protection for biodiversity conservation. Second, not all ecoregions are equal, and those that are widely distributed might be less of a global priority to protect than those that are restricted to one or a few countries. One requirement of Aichi Target 11 is that areas important for biodiversity should be a conservation priority. We thus could introduce a weighting system that reflects the desirability of each ecoregion for protection; the same is true for ecological relevance.

The two PE metrics assess the results of two potentially very different policies: should we expect countries to protect a set percentage of each habitat present within their borders or should we expect them to protect a set amount of each habitat? In other words, while representation is a key goal, there are at least two interpretations. Both policies have advantages and drawbacks. For example, protecting a fixed percentage of each habitat means that large habitats will receive more protection, in terms of total surface area. In practice, this results in a protection bias towards more abundant habitats. Protecting a set amount of each habitat ensures an equitable area of each is placed under protection. In practice, to achieve a high PE value, this amount is dictated by whichever habitat has the smallest area, creating a bias towards sparse habitats. The ecological implications of favouring one PE metric over the other over time can be drastic, and are important to understand and acknowledge. Favouring one or the other and aiming to improve that PE score with each protected area decision can end up shaping the reserve network in very different ways. Let us consider two extreme cases. If, on one hand, the smallest habitats within a country are more “desirable” (e.g. globally rare or endemic, or providing more ecosystem value), then their protection and representation is maximised by protecting a fixed area amount of all habitats (i.e. obtaining a larger PE_*p*_ score). Conversely, if the largest habitats are more “desirable” to the decision-makers, then protecting a set percentage can maximize their protection and the country’s ecological representation as measured by PE_*f*_.

By having two variants of the PE metrics, there may be incentive for countries to “game the system” by presenting the PE metric yielding the highest value each time reporting is required. Indeed, it is theoretically possible to increase proportional PE and decrease fixed-area PE, and vice-versa. A country as large as Australia could protect 100 km^2^ of each ecoregion, thus scoring PE_*f*_ = 1, but effectively protecting very little of its area. Alternatively, it could only protect 100% of the most abundant ecoregion, possibly millions of km^2^, and score PE_*f*_ = 0 and PE_*p*_ = 0 because there would be no ecological representation. Neither of these alternatives is ideal as ecological representation aims to protect the functional advantage of a diverse environment, but protecting too little of everything achieves no biodiversity benefits. It is important, for transparency and accountability, but also comparability, to calculate and report on the same metric(s) over time. If only one version of the metric is chosen, the reporting needs to be consistent i.e. use the proportional PE_*p*_ every time to measure the impact of decisions as PE_*p*_ values are comparable between each other’s, but not with PE_*f*_ values. However, we recommend calculating both metrics rather than choosing only one for reporting in an international policy context, in order to make results comparable between countries.

Given the percent area target of CBD Aichi Target 11, the proportional PE metric is the most appropriate to specifically report against it. Nevertheless, any given country that calculates PE_*p*_ across ecoregions and gets a perfect score of 1, only proves that it is meeting the equal representation goal as it could be only protecting 1% of each ecoregion; this is why mean *p*_*i*_*/a*_*i*_ (average percent protected) is also needed to give context to the PE score.

We acknowledge that some of the metrics already available in the conservation literature to measure community diversity could be adapted to the problem of PA representation. To illustrate, imagine that the set of ecoregions within a country is equivalent to a species community, and the aim is to measure components of its diversity. The equivalent of species richness is simply the count of ecoregions, species abundance is the amount of each ecoregion that is under protection, and an individual is a unit of a protected ecoregion, e.g. 1 ha. Could well-known diversity metrics be used to describe the PA network? For example, the Shannon-Wiener index is an often-used measure of diversity, although it truly measures entropy [29], which quantifies the uncertainty in predicting the “identity” of an individual that is taken at random from the dataset. Here, the individual identity is to which the ecoregion belongs. Similar to our PE metrics, if all abundance is concentrated in one type (i.e. all protected area is part of one ecoregion), and the other types are very rare (even if there are many of them), the entropy approaches zero. The issue with Shannon-Wiener, however, is that it is not bounded by an upper limit, which renders comparison between countries difficult. Another common diversity index is the Gini-Simpson coefficient, which is not an entropy but a probability [30]; bounded between 0 and 1, it represents the probability that two individuals picked up at random are not from the same species or ecoregion. The coefficient is zero when one ecoregion dominates and it is one when ecoregions are uniformly distributed, the probability of selecting each ecoregion is equal. In practice, however, it is a measure of whether one ecoregion is more dominant than others in the PA network [31]. While informative, the Gini-Simpson index measures a different aspect than the PE metrics, i.e. the evenness of ecoregions across the PA network.

### Management recommendations

In order to apply the PE metrics to assess representation inside a reserve network, decision-makers should observe a systematic procedure:

1. Define their objective: what do they want to achieve? For example, the objective may be to meet CBD targets such as Aichi Target 11.
2. Define specific parameters that will remain the same over time: What do they want to measure (e.g. ecoregions and/or specific habitat type)? At what spatial scale (e.g. regional and/or national) and temporal scale (e.g. annually or less often)?
3. Calculate the PE metrics for baseline reference (ProtectEqual package, [19]), which will be used for future comparison and assessing the impact of decisions. This choice must be clearly justified, as it is possible for PE to increase by chance as PA network expand, without representation being an explicit goal [32].
4. At every reporting time-step, if the PA network has changed, managers should recalculate both metrics to assess progress towards the set objective. PE metrics can also be used as part of cost-benefit analyses to identify the most cost-efficient actions for a reserve network; benefit is measured as the improvement in PE and cost is a function of which area is being protected.

### Conclusion

There is no clear definition of what good ecological representation is inside PAs. As a starting point, using proportional and fixed area targets for all conservation features present is the most reasonable conceptual approach that is also well established amongst managers. Our proposed metrics can assess how well each country performs against both measures of representation, and allow for transparent comparison. There needs to be further research into what sort of ecological representation yields the highest biodiversity outcomes, and how these targets can be integrated into international agreements. In this paper, we suggest several potential modifications to the PE metrics, which would account for a finer scale evaluation of the biodiversity outcomes of PAs.

## Supporting information

S1 Appendix A short primer on *ProtectEqual* package. (DOCX)

S2 Appendix Equations to calculate proportional and fixed area PE. (DOCX)

S1 Table Protection Equality (PE_*p*_) under perfect inequality calculated for *N* ecoregions. We expect PE to be equal to 0 but when N is small, PE is >>0. Similar results are obtained for fixed-area PE (PE_*f*_).
(DOCX)

S1 Fig Illustration of the bias in the Protection Equality metric. When *N* is small (*N* = 2, 3, 10 and 100), perfect inequality does not equal 0 as it should, giving rise to the need of a correction factor for small *N*.
(TIF)

S2 Fig Difference in PE between scenario 1 and scenario 2 as a function of the coefficient of variation of a_*i*_, and the number of ecoregions *N*. (TIF)
