# Supplementary material for: Methods for calculating Protection Equality for conservation planning
Source: PLoS One. 2017 Feb 15;12(2):e0171591. doi: 10.1371/journal.pone.0171591 (PMC5310882; doi:10.1371/journal.pone.0171591)
Supplement: S1 Appendix — (DOCX) [file pone.0171591.s001.docx]

**Appendix S1. A short primer on *ProtectEqual* package**

The backbone of the ProtectEqual () package in R is the function **pe**. It takes four arguments: the data as a list of pi and ai, the version of PE calculated as either “proportional” or “fixed”, a TRUE/FALSE statement about whether an automatic plot should be produced, and a TRUE/FALSE statement about whether the corrected PEc should be used. R package uploaded to GitHub < <https://github.com/AChauvenet/ProtectEqual>>.

library(ProtectEqual)
N<-10 # number of regions
set.seed(1)
data<-data.frame(ai=rep(0,N),pi=rep(0,N))

# generate N ai values
data$ai<-round(runif(N,1, 2000000),0)
for (p in 1:N)
{

# generates N pi values that are never bigger than total area

(max is corresponding ai)
set.seed(15+p)
data$pi[p]<-round(runif(1,0,data$ai[p]),0)
}

# runs the PE function to calculate “proportional” and “fixed Protection Equality
pe(data,"proportional",plot_pe=TRUE, correct=TRUE)
pe(data,"proportional",plot_pe=TRUE, correct=TRUE)$PE
pe(data,"fixed")

The function **pe** outputs seven arguments: ‘PE’ is the Protection Equality of the region of interest; ‘version’ is the version of PE used for the calculations; ‘N’ is the number of ecoregions used to calculate PE; ‘Geom’ tests that no data point crosses over the line of perfect equality; ‘Slope’ tests that the slopes are in increasing order; ‘Sorted_stand_xaxis’ and ‘Sorted_stand_yaxis’ are the standardized cumulative x and y values to plot the PE curve.
