# Supplementary material for: Methods for calculating Protection Equality for conservation planning
Source: PLoS One. 2017 Feb 15;12(2):e0171591. doi: 10.1371/journal.pone.0171591 (PMC5310882; doi:10.1371/journal.pone.0171591)
Supplement: S2 Appendix — (DOCX) [file pone.0171591.s002.docx]

**Appendix S2. Equations to calculate proportional and fixed area PE**

1. Fixed area protection (PE*_np_*)

If the level of protection of ecoregions *y_i_* is measured as the absolute amount of protection using *p_i_* (in ha):

$$y_{i}=\sum_{j=1}^{i} p_{j}$$

hence,

$$y_{N}=\sum_{i=1}^{N} p_{i}$$

and the denominator becomes:

$$U+V=\frac{1}{2}\times\sum_{i=1}^{N} p_{i}$$

We can approximate U as the sum of the areas of triangles and rectangles under the curve of protection (delimited by dashed lines in Figure 1):

$$\sum area triangles= \sum_{i=1}^{N} p_{i}\times\frac{1}{N}\times\frac{1}{2}$$

$$\sum area rectangles= \sum_{i=1}^{N-1} p_{i}\times\frac{1}{N}\times\left( N-i \right)$$

The nominator then becomes:

$$U=\frac{1}{N}\times\left( \frac{1}{2}\sum_{i=1}^{N} p_{i}+ \sum_{i=1}^{N-1} p_{i}\times\left( N-i \right) \right)$$

1. Proportional protection (PE*_p_*)

If the level of protection of ecoregions *y_i_* is measured as the proportion of the ecoregion protected using *p_i_* / *a_i_*:

$$y_{i}=\sum_{j=1}^{i} \frac{p_{j}}{a_{j}}$$

hence,

$$y_{N}=\sum_{i=1}^{N} \frac{p_{i}}{a_{i}}$$

and the denominator becomes:

$$U+V=\frac{1}{2}\times\sum_{i=1}^{N} \frac{p_{i}}{a_{i}}$$

Then,

$$\sum area triangles= \sum_{i=1}^{N} \frac{1}{N}\times\frac{p_{i}}{a_{i}}\times\frac{1}{2}$$

$$\sum area rectangles= \sum_{i=1}^{N-1} \frac{1}{N}\times\frac{p_{i}}{a_{i}}\times\left( N-i \right)$$

so,

$$U= \frac{1}{N}\times\left( \frac{1}{2}\sum_{i=1}^{N} \frac{p_{i}}{a_{i}}+ \sum_{i=1}^{N-1} \frac{p_{i}}{a_{i}}\times\left( N-i \right) \right)$$
