# Supplementary material for: Methods for calculating Protection Equality for conservation planning
Source: PLoS One. 2017 Feb 15;12(2):e0171591. doi: 10.1371/journal.pone.0171591 (PMC5310882; doi:10.1371/journal.pone.0171591)
Supplement: S1 Table — We expect PE to be equal to 0 but when N is small, PE is >>0. Similar results are obtained for fixed-area PE (PEf). (DOCX) [file pone.0171591.s003.docx]

**Table S1.** Protection Equality (PE*_p_*) under perfect inequality calculated for *N* ecoregions. We expect PE to be equal to 0 but when N is small, PE is >>0. Similar results are obtained for fixed-area PE (PE*_f_*)

| ***N*** | **PE** |
| --- | --- |
| 2 | 0.5 |
| 3 | 0.33 |
| 4 | 0.25 |
| 5 | 0.2 |
| 6 | 0.17 |
| 7 | 0.14 |
| 8 | 0.12 |
| 9 | 0.11 |
| 10 | 0.1 |
| 11 | 0.09 |
| 12 | 0.08 |
| 13 | 0.08 |
| 14 | 0.07 |
| 15 | 0.07 |
| 16 | 0.06 |
| 17 | 0.06 |
| 18 | 0.06 |
| 19 | 0.05 |
| 20 | 0.05 |
| 21 | 0.05 |
| 22 | 0.05 |
| 23 | 0.04 |
| 24 | 0.04 |
| 25 | 0.04 |
| 26 | 0.04 |
| 27 | 0.04 |
| 28 | 0.04 |
| 29 | 0.03 |
| 30 | 0.03 |
| 31 | 0.03 |
| 32 | 0.03 |
| 33 | 0.03 |
| 34 | 0.03 |
| 35 | 0.03 |
| 36 | 0.03 |
| 37 | 0.03 |
| 38 | 0.03 |
| 39 | 0.03 |
| 40 | 0.02 |
| 41 | 0.02 |
| 42 | 0.02 |
| 43 | 0.02 |
| 44 | 0.02 |
| 45 | 0.02 |
| 46 | 0.02 |
| 47 | 0.02 |
| 48 | 0.02 |
| 49 | 0.02 |
| 50 | 0.02 |
| 51 | 0.02 |
| 52 | 0.02 |
| 53 | 0.02 |
| 54 | 0.02 |
| 55 | 0.02 |
| 56 | 0.02 |
| 57 | 0.02 |
| 58 | 0.02 |
| 59 | 0.02 |
| 60 | 0.02 |
| 61 | 0.02 |
| 62 | 0.02 |
| 63 | 0.02 |
| 64 | 0.02 |
| 65 | 0.02 |
| 66 | 0.02 |
| 67 | 0.01 |
| 68 | 0.01 |
| 69 | 0.01 |
| 70 | 0.01 |
| 71 | 0.01 |
| 72 | 0.01 |
| 73 | 0.01 |
| 74 | 0.01 |
| 75 | 0.01 |
| 76 | 0.01 |
| 77 | 0.01 |
| 78 | 0.01 |
| 79 | 0.01 |
| 80 | 0.01 |
| 81 | 0.01 |
| 82 | 0.01 |
| 83 | 0.01 |
| 84 | 0.01 |
| 85 | 0.01 |
| 86 | 0.01 |
| 87 | 0.01 |
| 88 | 0.01 |
| 89 | 0.01 |
| 90 | 0.01 |
| 91 | 0.01 |
| 92 | 0.01 |
| 93 | 0.01 |
| 94 | 0.01 |
| 95 | 0.01 |
| 96 | 0.01 |
| 97 | 0.01 |
| 98 | 0.01 |
| 99 | 0.01 |
| 100 | 0.01 |
