# Supplementary figures and images for: Methods for calculating Protection Equality for conservation planning
Source: PLoS One. 2017 Feb 15;12(2):e0171591. doi: 10.1371/journal.pone.0171591 (PMC5310882; doi:10.1371/journal.pone.0171591)

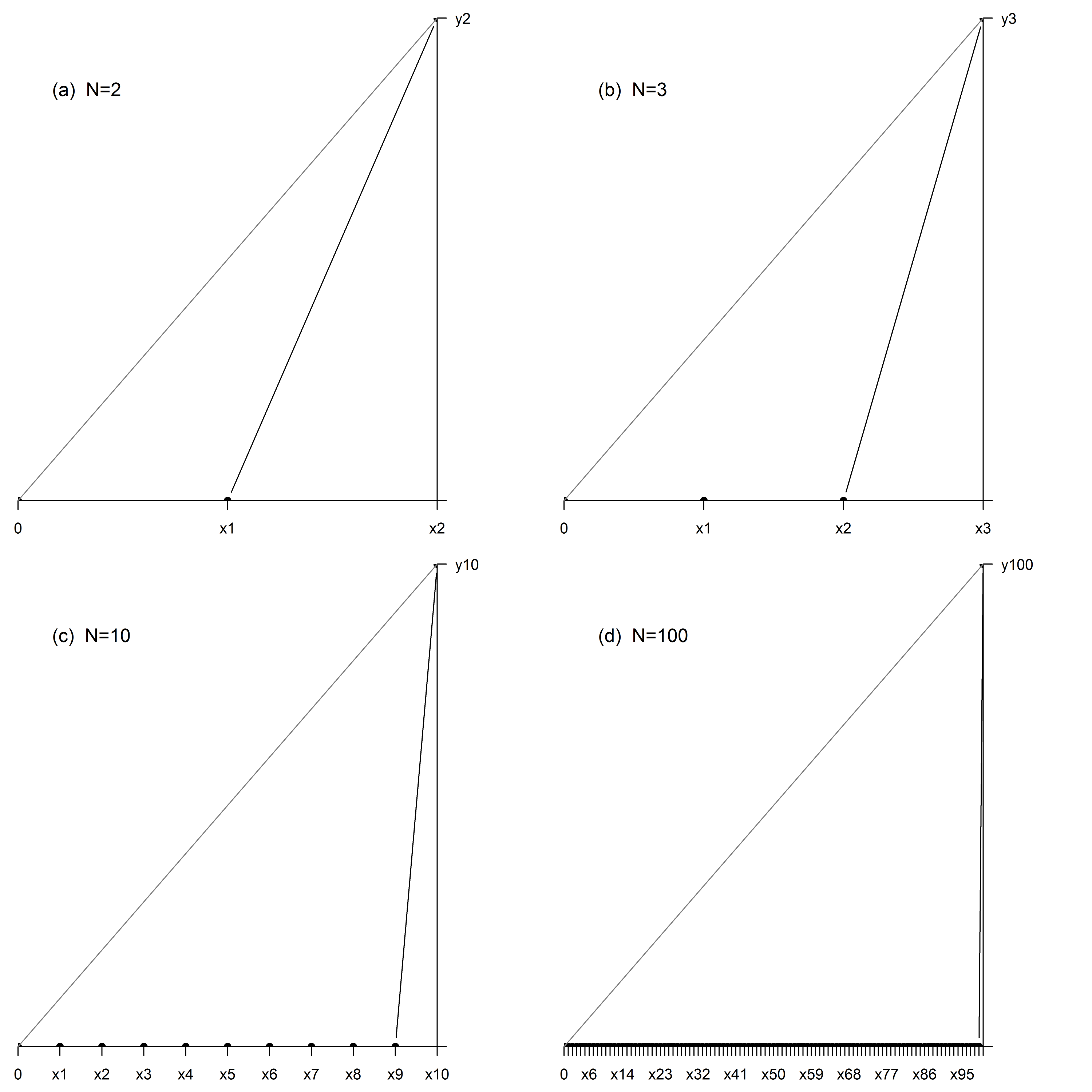

Supplement: S1 Fig — When N is small (N = 2, 3, 10 and 100), perfect inequality does not equal 0 as it should, giving rise to the need of a correction factor for small N. (TIF) [file pone.0171591.s004.tif]

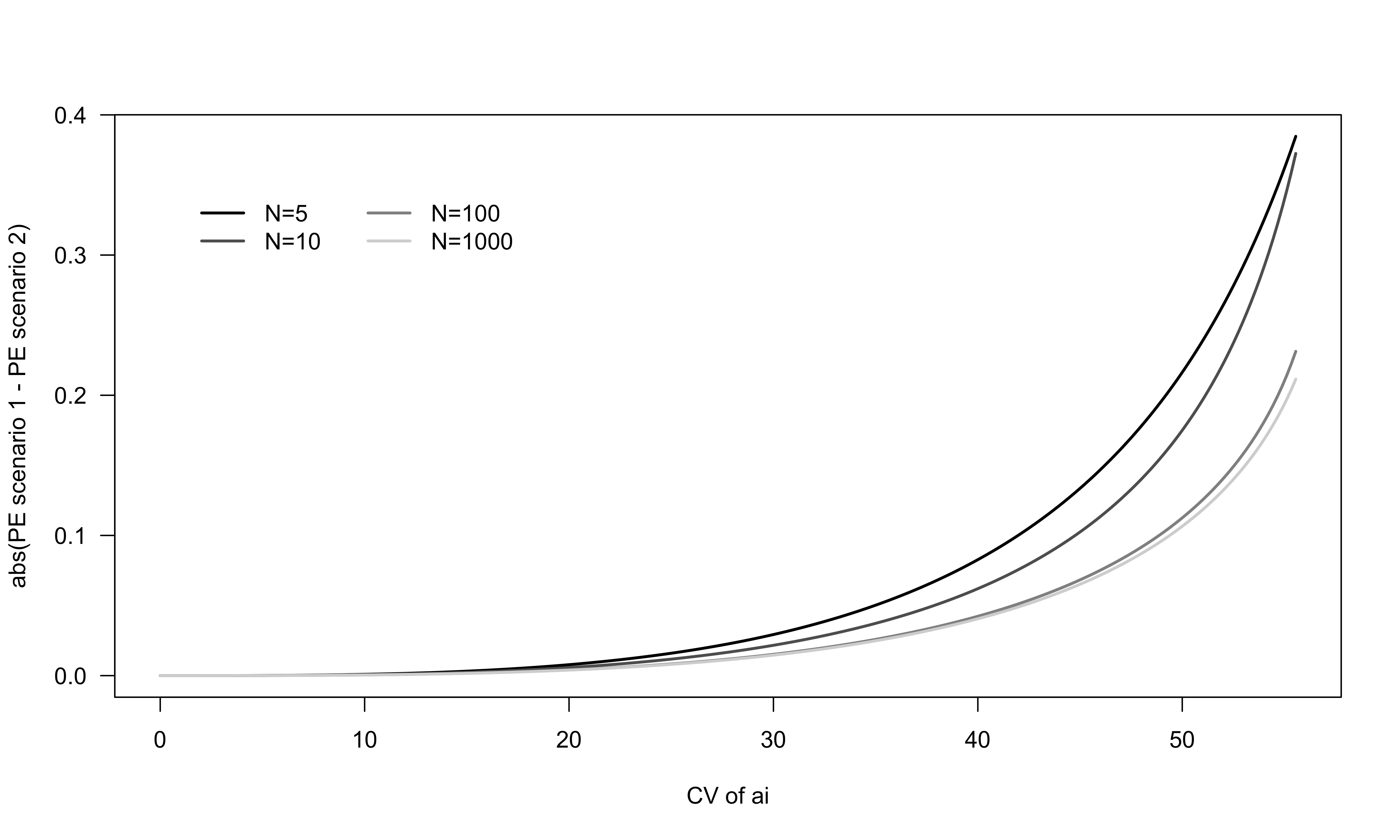

Supplement: S2 Fig — (TIF) [file pone.0171591.s005.tif]
